# GCN5 Potentiates Glioma Proliferation and Invasion via STAT3 and AKT Signaling Pathways

**DOI:** 10.3390/ijms160921897

**Published:** 2015-09-10

**Authors:** Kun Liu, Qing Zhang, Haitao Lan, Liping Wang, Pengfei Mou, Wei Shao, Dan Liu, Wensheng Yang, Zhen Lin, Qingyuan Lin, Tianhai Ji

**Affiliations:** 1 Department of Pathology, Affiliated Chenggong Hospital, Xiamen University, Xiamen 361000, China; 2 Chinese People’s Liberation Army No. 174 Clinical College, Anhui Medical University, Xiamen 361000, China; 3 Department of Oncology, Sichuan Academy of Medical Sciences & Sichuan Provincial People’s Hospital, Chengdu 610072, China

**Keywords:** GCN5, cell proliferation, cell invasion, STAT3

## Abstract

The general control of nucleotide synthesis 5 (GCN5), which is one kind of lysine acetyltransferases, regulates a number of cellular processes, such as cell proliferation, differentiation, cell cycle and DNA damage repair. However, its biological role in human glioma development remains elusive. In the present study, we firstly reported that GCN5 was frequently overexpressed in human glioma tissues and GCN5 was positively correlated with proliferation of cell nuclear antigen PCNA and matrix metallopeptidase MMP9. Meanwhile, down-regulation of GCN5 by siRNA interfering inhibited glioma cell proliferation and invasion. In addition, GCN5 knockdown reduced expression of p-STAT3, p-AKT, PCNA and MMP9 and increased the expression of p21 in glioma cells. In conclusion, GCN5 exhibited critical roles in glioma development by regulating cell proliferation and invasion, which suggested that GCN5 might be a potential molecular target for glioma treatment.

## 1. Introduction

Glioma is the most common malignant brain tumor and usually occurs with high morbidity, high recurrence rate and high mortality, among which astrocytic glioma accounts for the largest subgroup [1,2]. Even with recent advances in cancer treatment, like surgical resection or chemoradiotherapy, it is still extremely difficult to cure glioma because of its high invasion ability. The median survival time of patients with glioma such as glioblastoma multiforme (GBM) is only 12–15 months [3,4,5]. Thus, a better understanding of the molecular mechanisms underlying glioma tumorigenesis and identifying more effective biomarkers are conducive to the development of targeted therapy.

Histone acetylation is a major kind of protein modification method and plays a pivotal role in regulating cellular processes [6,7,8,9]. Histone acetylation plays a central role in establishing an active chromatin environment for transcriptional regulation. The balance between histone acetylation and deacetylation is maintained in cells by histone lysine acetyltransferases (KATs) and histone deacetylases (HDACs). Previous studies have shown that lysine acetyltransferases are involved in cancer development, such as breast cancer and hepatocellular carcinoma [10,11,12]. Hence, new KAT inhibitors are investigated and represent useful tools for cancer prevention and therapy [13].

GCN5, the first identified transcriptional-related KAT, is one important catalytic component of a transcriptional regulatory complex. GCN5 is reported to play important roles in cell proliferation, differentiation, cell cycle, and DNA damage repair [14,15,16]. Furthermore, studies have proved that GCN5 is involved in certain cancer development, such as non-small cell lung cancer and breast cancer [16,17,18]. While no studies have assessed the role of GCN5 in glioma, which promotes our interest in investigating its biological role in glioma.

In the present study, to elucidate the biological role of GCN5 in glioma tumorigenesis, we evaluated GCN5 expression in 15 pairs of human glioma tissues and found that GCN5 was frequently overexpressed in human glioma tissues. In addition, down-regulation of GCN5 in glioma cell inhibited cell proliferation, colony formation and invasion ability. Furthermore, down-regulation of GCN5 could decrease the expression of p-STAT3, p-AKT, PCNA, and MMP9 and increase the expression of p21. The results, support that GCN5 would provide a novel therapeutic target for human glioma.

## 2. Results

### 2.1. GCN5 Was Frequently Overexpressed in Human Glioma Tissues

**Figure 1:**
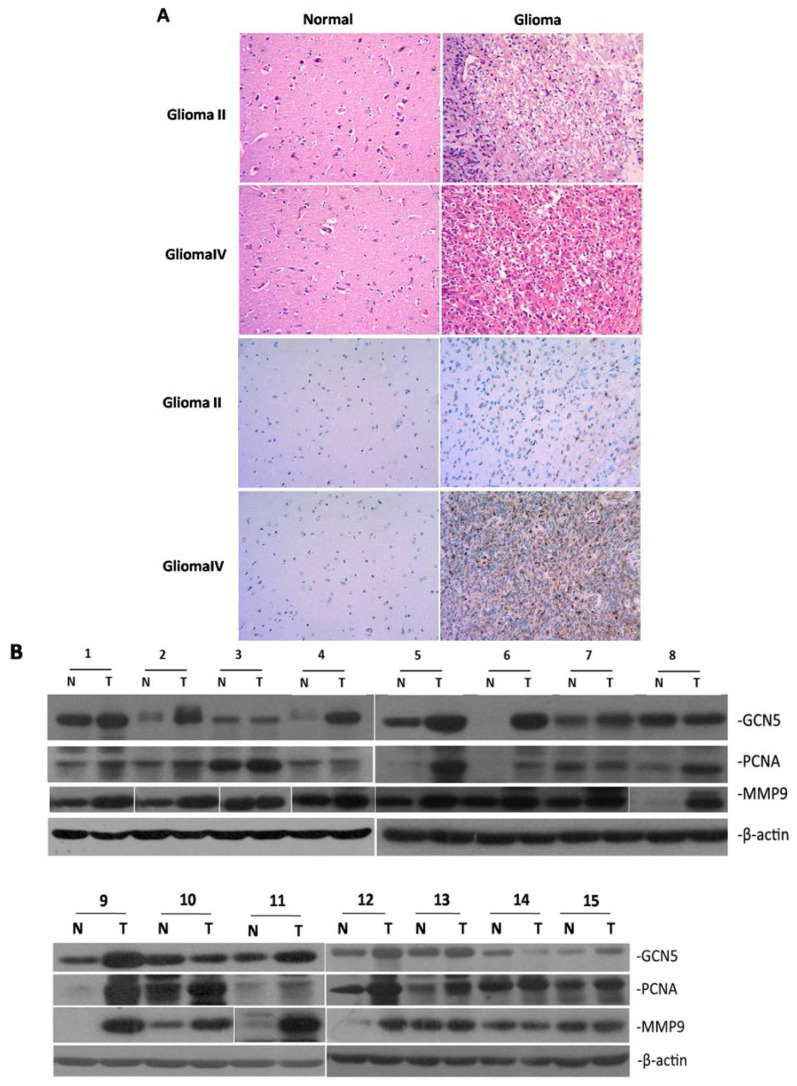

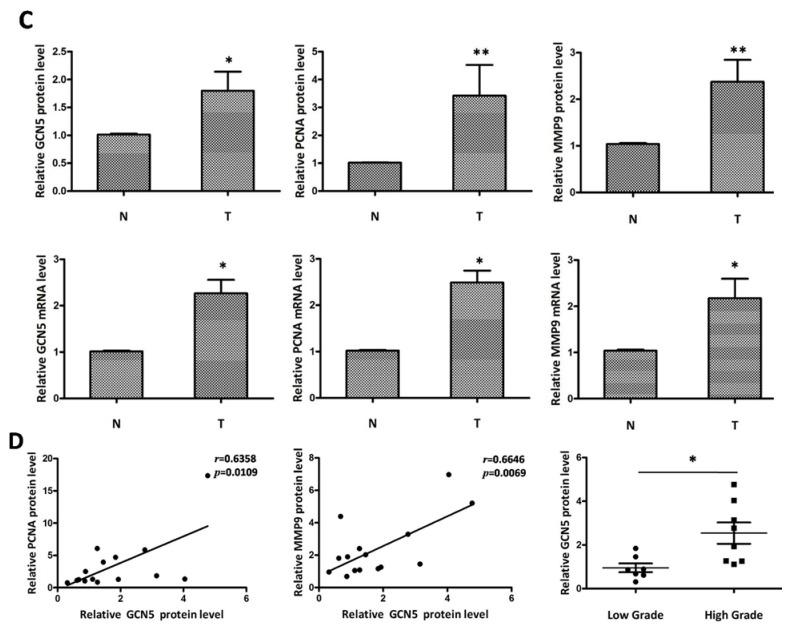
GCN5 was frequently overexpressed in human glioma tissues. (**A**) Glioma and adjacent non-tumor tissue were pathologically diagnosed by HE staining; GCN5 expression in gliomas was determined by IHC (Magnification ×200); (**B**) GCN5, PCNA and MMP9 protein level were determined in 15 (Number1-15) pairs of glioma tissues by Western blot (N: Non-tumor tissue; T: Tumor tissue); (**C**) GCN5, PCNA and MMP9 were overexpressed in glioma tissues compared with adjacent non-tumor tissues both at protein and mRNA level; and (**D**) GCN5 protein level was positively associated with PCNA and MMP9 level, respectively; GCN5 protein level in high-grade group was much higher than in low-grade group tissues (* *p* < 0.05; ** *p* < 0.01).

Gliomas used in this study were firstly diagnosed by HE staining as shown in Figure 1A. Characteristics of glioma patients were summarized in Table S1. We investigated GCN5 expression in 15 pairs of glioma and adjacent non-tumor tissues by immunohistochemistry (IHC). IHC result indicated that GCN5 expression was negative in adjacent normal brain tissue. Weak cytoplasmic GCN5 staining was observed in low-grade glioma and strong cytoplasmic GCN5 expression was observed in high-grade glioma, as determined in Figure 1A. To further determine the expression of GCN5 in human glioma tissues, Western blot and RT-PCR were performed to assess the level of GCN5 expression in 15 pairs of glioma and adjacent non-tumor tissues. Both Western blot and RT-PCR analysis displayed that GCN5 was frequently upregulated in glioma compared with the adjacent non-tumor tissue (*p* < 0.05 and *p* < 0.05; Figure 1B,C), suggesting a possible involvement of GCN5 in the development of glioma.

We also examined PCNA and MMP9 expression in glioma tissues by using Western blot and RT-PCR. The result showed that PCNA and MMP9 were also upregulated in glioma tissues compared with non-tumor tissues both at mRNA and protein level (Figure 1C). To further evaluate the relationship between GCN5 with PCNA and MMP9 expression, we performed the quantitative analysis. Statistical analysis indicated that the protein level of GCN5 was positively correlated with the protein level of PCNA and MMP9, respectively (*p* < 0.05 and *p* < 0.01; Figure 1D). This result indicated that GCN5 may regulate glioma cell proliferation and invasion.

To investigate whether GCN5 expression was associated with glioma grade, we divided these 15 pairs of glioma patients into two groups: low-grade group (Grade I–II) and high-grade group (Grade III–IV). As determined in Figure 1D, GCN5 expression in high-grade group was much higher than in low-grade group (*p* < 0.05). Thus, these results implied that GCN5 might be a novel prognostic marker for glioma grade.

All in all, our above results showed that GCN5 was upregulated in human glioma tissues and was associated with PCNA and MMP9 expression. GCN5 was also associated with glioma grade. We hypothesized that GCN5 might be involved in the development of glioma.

**Figure 2:**
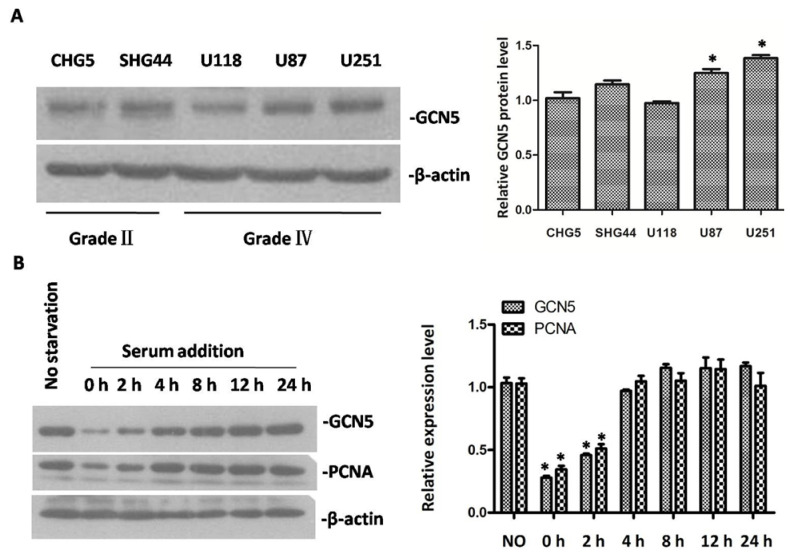
GCN5 expression in glioma cell lines and cell cycle progression. (**A**) The expression of GCN5 in five glioma cell lines was determined by Western blot; and (**B**) GCN5 and PCNA expression pattern in serum starvation and releasing model experiment (* *p* < 0.05, compared with NO starvation).

### 2.2. The Expression of GCN5 in Glioma Cell Lines and Cell Cycle Progression

To verify the role of GCN5 in glioma development, we chose five glioma cell lines, CHG5, SHG44 (Grade II), U118, U251, and U87 (Grade IV), for the following research. Data indicated that GCN5 was highly expressed in these five cell lines (Figure 2A). U251 and U87 cells expressed a higher level of GCN5 compared with CHG5, so we chose these two cell lines for our following study.

Since GCN5 expression was associated with PCNA expression in glioma tissues (Figure 1D), we performed a serum starvation and releasing model to detect GCN5 expression during cell cycle progression. As shown in Figure 2B, GCN5 expression became gradually increased with the passage of time, which was consistent with PCNA expression. These data indicated that GCN5 was involved in the cell cycle regulation.

### 2.3. Silencing GCN5 Expression in Glioma Cells

**Figure 3:**
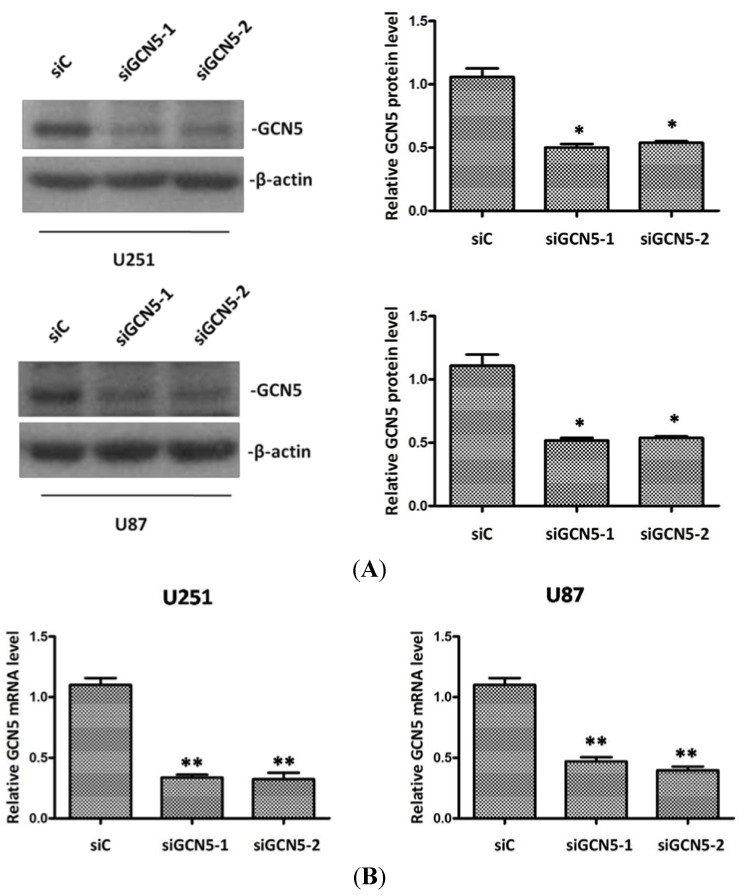
GCN5 expression was suppressed after siRNA treatment. (**A**) Western blot showed that GCN5 protein level was significantly decreased after siRNA treatment. (**B**) RT-PCR showed that GCN5 mRNA level was significantly downregulated after siRNA treatment (* *p* < 0.05; ** *p* < 0.01).

To further examine the biological functions of GCN5 in glioma, two different siRNAs against GCN5 were used to interfere GCN5 expression in U251 and U87. Forty-eight hours after transfection, Western bolt and RT-PCR were performed to determine GCN5 knockdown efficiency. Data showed that both protein and mRNA level of GCN5 were significantly decreased in siGCN5 group compared with control group (Figure 3A,B). These results implicated that we had succeeded in knocking down GCN5 expression.

### 2.4. GCN5 Knockdown Suppressed Cell Proliferation and Colony Formation

**Figure 4:**
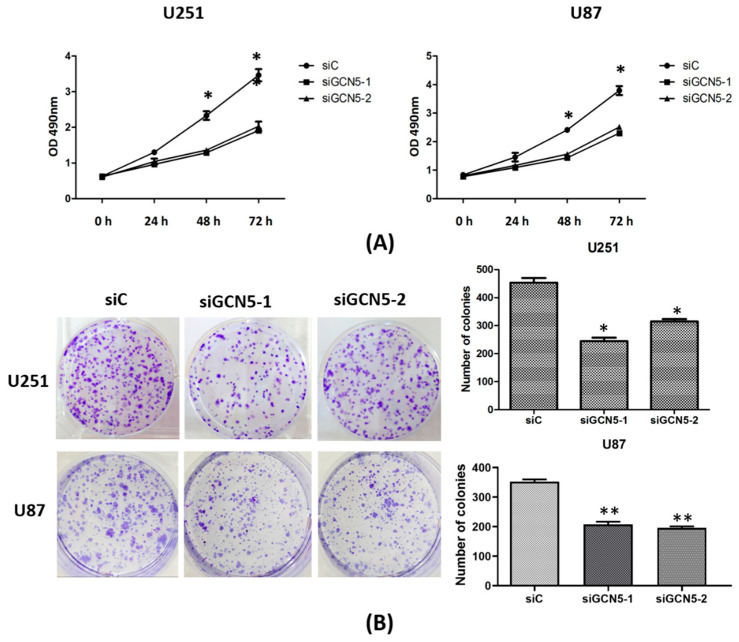
GCN5 knockdown suppressed cell proliferation and colony formation ability. (**A**) MTS assay indicated that GCN5 knockdown suppressed cell growth rate in U251 and U87 cell lines; (**B**) Colony formation assay showed that GCN5 knockdown suppressed cell colony formation ability in U251 and U87 cell lines (* *p* < 0.05; ** *p* < 0.01).

To examine whether GCN5 affects glioma proliferation, MTS assay and colony formation assay were performed to determine cell growth rate and colony formation ability. Twenty-four hours after transfection, cells were seeded into a 96-well plate (4000 per well). Every 24 h, 20 μL of MTS was added into each well and then cells were incubated for 4 h at 37 °C. Finally, the absorbance was measured at 490 nm using a microplate reader. MTS assay showed that GCN5 knockdown significantly decreased cell growth rate of siGCN5 group compared with control group (Figure 4A). Forty-eight hours after transfection, cells were counted and seeded (1000 per dish) into a 6-well dish and incubated in 37 °C. Fresh culture medium was replaced every 2 days. Three weeks later, cells were washed with PBS and stained with Gimesa. Colonies with more than 50 cells were counted. Colony formation assay showed that GCN5 knockdown significantly reduced the number of cell colonies formed compared with the control group (Figure 4B). These data indicated that GCN5 was vital for the glioma proliferation.

### 2.5. GCN5 Knockdown Inhibited Cell Invasion

**Figure 5:**
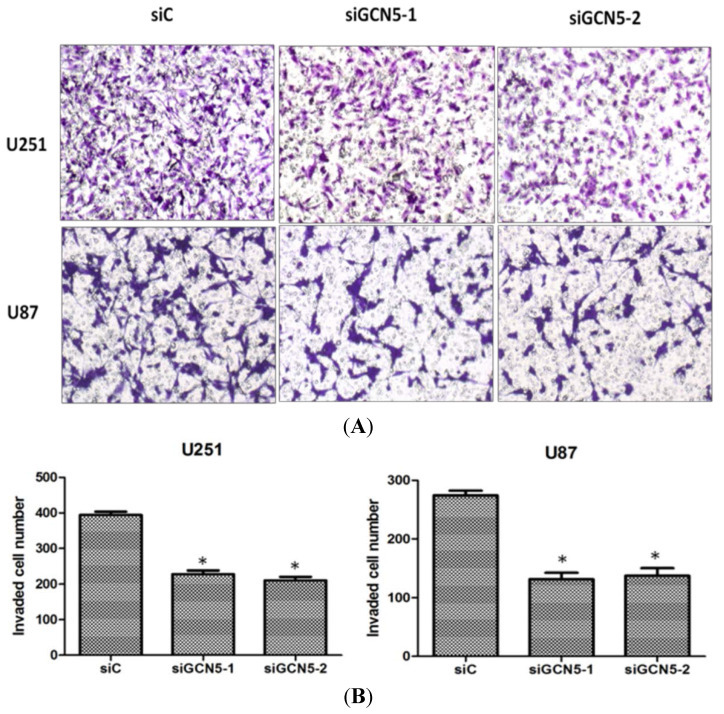
GCN5 knockdown inhibited cell invasion. (**A**) Downregulation of GCN5 after siRNA treatment inhibited cell invasion in U251 and U87 cell lines (Magnification× 100); and (**B**) Quantification analysis showed that GCN5 knockdown could suppress cell invasion in U251 and U87 cell lines (* *p* < 0.05).

On account that glioma is a type of malignant brain tumor with high invasion ability, we determined whether GCN5 had an impact on cell invasiveness. Then transwell assay was performed to study the invasive ability of glioma cells. Twenty-four hours after transfection, cells were counted and then seeded into interior. Twenty-four hours later, non-invading cells in the interior of the insert were removed by a cotton swab; the invasive cells on the lower surface of the insert were stained with Gimesa for 20 min and counted under a microscope. As shown in Figure 5, GCN5 knockdown could significantly suppress glioma cell invasiveness compared with control group (Figure 5A,B).

### 2.6. GCN5 Knockdown Decreased the Expression of p-STAT3, p-AKT, PCNA, MMP9 and Increased the Expression of p21 Expression in Glioma Cells

**Figure 6:**
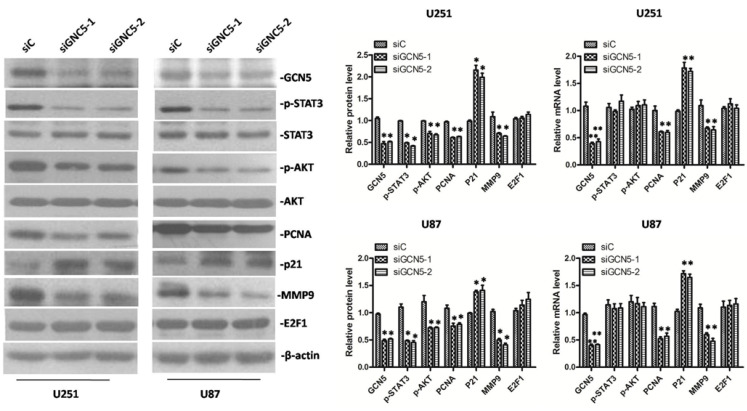
GCN5 knockdown decreased the expression of p-STAT3, p-AKT, PCNA, and MMP9 and increased the expression of p21 in glioma cells (* *p* < 0.05; ** *p* < 0.01).

It has been reported that GCN5 can promote cell proliferation through enhancing E2F1 in non-small cell lung cancer [16]. Here, we also examined the protein level of E2F1 in GCN5 knockdown cells but no change of E2F1 was detected (Figure 6). To further explore the mechanism by which downregulation of GCN5 inhibited glioma cell proliferation and invasion, a number of genes that regulate cell molecular signaling were detected in siGCN5 cells. Compared with control group, we found that GCN5 knockdown observably decreased the expression of p-STAT3, p-AKT, PCNA and increased the expression of p21 in siGCN5 group (Figure 6). These findings indicated that GCN5 promoted cell proliferation and invasion at least in part through enhancing these signaling pathways rather than E2F1 pathway in glioma.

## 3. Discussion

Histone acetylation is vital in establishing an active chromatin environment for transcriptional regulation. Previous studies have shown that lysine acetyltransferases play complicated roles in cancer development [6,7,8,9,10,11,12]. Much evidence has shown that GCN5 plays important roles in regulating cell proliferation, differentiation, cell cycle and DNA damage repair. It is reported that GCN5 regulates cancer development in several cancers such as non-small cell lung cancer and breast cancer [15,16]. However, the biological function of GCN5 in glioma is still unclear. In the present study, we found that GCN5 was highly expressed in glioma tissues compared with adjacent non-tumor tissues both at protein and mRNA level. We also reported that GCN5 expression was correlated with PCNA and MMP9 in glioma tissues. Furthermore, GCN5 expression level in high-grade glioma tissues was much higher than in low-grade glioma tissues. In the serum starvation and releasing model experiment, GCN5 became gradually increased consistent with PCNA level. All these results indicated GCN5 was involved in cell development. In U87 and U251 cell lines, by knocking down GCN5 expression with siRNA interfering, we revealed that GCN5 down-regulation could effectively suppress cell growth rate, colony formation and cell invasion ability.

Signal transducer and activator of transcription 3 (STAT3) is reported to have a diversity of biological functions in regulating cell proliferation, differentiation, apoptosis, inflammation, oncogenesis and angiogenesis in many kinds of tumors including glioma [19,20,21,22,23,24]. Moreover, studies have showed that AKT pathway regulate various cell functions, such as angiogenesis, migration and survival in glioma [25]. We therefore examined if GCN5 can regulate p-STAT3 and p-AKT expression. By knocking down GCN5 expression with siRNA interfering, we found that GCN5 knockdown can significantly downregulate p-STAT3 and p-AKT, indicated that GCN5 can regulate cell proliferation and invasion at least in part through STAT3 and AKT pathways. In non-small cell lung cancer, GCN5 was proved to be recruited by E2F1 to E2F1-binding sites to regulate downstream target expression [16], but it turned out that down-regulation of GCN5 did not cause significant change of E2F1 expression in glioma cells. This result indicates that the interaction between GCN5 and E2F1 promoter region in glioma may not exist. To further explore whether there is any interaction between GCN5 and E2F1, Co-IP and X-ChIP experiments are needed to determine this claim. In GCN5 knockdown cells, we also revealed that down-regulation of GCN5 resulted in a significant decrease in the expression of PCNA, MMP9 and an increase in the expression of cell cycle inhibitor p21, which are involved in cell proliferation and invasion. In addition, we also wanted to know the effect of GCN5 in low-grade glioma cell. GCN5 was downregulated in CHG5 cell, MTS assay was used to determine cell growth. We also detected p-STAT3, p-AKT, MMP9, PCNA and p21 in CHG5 cell, which is shown in Figure S1A,B. Data showed that GCN5 knockdown could inhibit cell growth and decrease the expression of p-STAT3, p-AKT, MMP9, and PCNA but increase the expression of p21 in CHG5 cell.

In conclusion, we proved that GCN5 was overexpressed in human glioma tissues. GCN5 can regulate cell proliferation and invasion partly through enhancing STAT3 and AKT signaling pathways. These findings implicated that GCN5 could be a potential target for glioma therapy.

## 4. Materials and Methods

### 4.1. Glioma Tissues

Glioma tissues were collected from 15 patients who were diagnosed with glioma and underwent curative surgery at Affiliated Chenggong Hospital. Informed consents were obtained from all patients and family members and this study was approved by the Ethical Committee of Affiliated Chenggong Hospital. All tissues were immediately frozen in liquid nitrogen and stored at −80 °C until use.

### 4.2. Immunohistochemistry

Five-micrometer-thick sections were prepared from paraffin-embedded tissues. Immunostaining was performed by the two-step Elivision plus kit (MaiXin, Fuzhou, China). The sections were deparaffinized in xylene, rehydrated with graded alcohol, and then boiled in citrate buffer (pH 6.0) for 2 min in an autoclave. Next, 0.3% hydrogen peroxide was applied to block the endogenous peroxidase activity, and the sections were incubated with normal animal serum to reduce non-specific binding. Tissue sections were incubated with GCN5 antibody (1:150 dilution; Santa Cruze Biotechnology, Santa Cruze, CA, USA) for 2 h at room temperature. Rabbit immunoglobulin (at the same concentration as for the antigen-specific antibody) was used as a negative control. The staining was followed by incubation with polymer secondary antibodies (MaiXin, Fuzhou, China). The peroxidase reaction was developed with DAB (3,3-diaminobenzidine, tetrahydrochloride). Counterstaining was done with hematoxylin, and the sections were dehydrated in alcohol before mounting.

### 4.3. Cell Culture and Transfection

The glioma cell lines CHG5 and SHG44 were kindly provided by Xiuwu Bian from Third Military Medical University, China. U87 and U118 cell lines were obtained from American Type Culture Collection (ATCC, Manassas, VA, USA). U251 cell line was obtained from China Center for Typical Culture Collection (CCTCC). Cells were cultured in DMEM (Invitrogen, Carlsbad, CA, USA) supplemented with 10% FBS (GEMINI, Woodland, CA, USA) and maintained in a humidified atmosphere at 37 °C with 5% CO_2_.

Two GCN5 siRNAs were synthesized from Invitrogen. Twenty-four hours prior to transfection, U87 and U251 cells were plated onto a 6-well plate at 70%–90% confluency. Cells were then transfected with siRNA at final concentration of 50 nM with the DharmaFECT reagent (Thermo Fisher Scientific, Lafayette, CO, USA) according to the manufacturer’s instrument. After 6 h of incubation at 37 °C, the transfection medium was replaced with 2 mL complete medium containing 10% FBS. Cells were collected for the following experiments at the indicated times.

### 4.4. MTS Assay

Twenty-four hours after transfection, cells were seeded into a 96-well plate (4000 per well). MTS assay (Promega, Madison, WI, USA) was used to determine relative cell growth. Every 24 h, 20 μL of MTS was added into each well and then cells were incubated for 4 h at 37 °C. Finally, the absorbance was measured at 490 nm using a microplate reader (Bio-Rad iMark, Hercules, CA, USA). All experiments were performed in triplicate.

### 4.5. Colony Formation Assay

Forty-eight hours after transfection, cells were counted and seeded (1000 per dish) into a 6-well dish and incubated in 37 °C. Fresh culture medium was replaced every 2 days. Three weeks later, cells were washed with PBS and stained with Gimesa (Millipore, Billerica, WI, USA). Colonies with more than 50 cells were counted. The colonies were manually counted using a microscope. All experiments were performed in triplicate.

### 4.6. Cell Invasion Assay

Cell invasion were assessed using the 24 well-plate transwell insert (Millipore, Billerica, MA, USA). In brief, 500 μL of prepared serum-free suspension of transfected cells (24 h after transfection) with a density of 1 × 10^5^ were added into the interior of each interior (8 μm pore size); 500 μL of medium containing 10% FBS was added to the lower chamber of the insert. Twenty-four hours after incubation in 5% CO_2_, non-invading cells in the interior of the insert were removed by a cotton swab; the invasive cells on the lower surface of the insert were stained with Gimesa for 20 min and counted under a microscope. All experiments were performed in triplicate.

### 4.7. Western Bolt Analysis

Total protein from tissue and cells was harvested in RIPA lysis buffer. After centrifugation at 12,000 rpm for 10 min, the protein concentration was measured by BCA protein assay kit (Pierce Appleton, WI, USA). Then, 30 μg of total protein was loaded onto SDS-PAGE gel. After electrophoresis, protein were transferred to PVDF membrane (Millipore) and incubated with blocking buffer for 60 min at room temperature and then incubated with primary antibody 4 °C overnight. Finally, proteins were detected with HRP-conjugated secondary antibody. The primary antibodies used were as followed: GCN5 (Santa Cruze), AKT (CST), p-AKT (CST), STAT3 (CST), p-STAT3 (CST), PCNA (CST), MMP9 (CST), E2F1 (Boster), P21 (CST), and β-actin (CST). Western blot quantitative analysis was performed by Scion Image software (Scion Corporation, Frederick, MD, USA).

### 4.8. RNA Extraction and RT-PCR Analysis

Total RNA was extracted from tissue and cells using Tripure Isolation Reagent (Roche, Indianapolis, IN, USA) according to the manufacturer’s instrument. cDNA was synthesized from 2 μg total RNA using the Transcriptor First Strand cDNA Synthesis Kit (Roche). RT-PCR was performed in triplicates with SYBR Green I Master (Roche). GAPDH was used as an internal control.

### 4.9. Statistical Analysis

Statistical analysis was performed using GraphPad Prism 5 (GraphPad Software, Inc., San Diego, CA, USA). Each experiment was performed at least three times. The data was expressed as mean ± SD. An unpaired Student’s *t*-test was used to determine the significant differences of all the results. The correlation between GCN5 with PCNA and MMP9 were analyzed using the Spearman test. *p* < 0.05 was considered statistically significant.

## Supplementary Materials

Supplementary materials can be found at https://www.mdpi.com/1422-0067/16/09/21897/s1.
