# Correction: Liu et al. GCN5 Potentiates Glioma Proliferation and Invasion via STAT3 and AKT Signaling Pathways. *Int. J. Mol. Sci.* 2015, *16*, 21897–21910

**DOI:** 10.3390/ijms26168068

**Published:** 2025-08-21

**Authors:** Kun Liu, Qing Zhang, Haitao Lan, Liping Wang, Pengfei Mou, Wei Shao, Dan Liu, Wensheng Yang, Zhen Lin, Qingyuan Lin, Tianhai Ji

**Affiliations:** 1 Department of Pathology, Affiliated Chenggong Hospital, Xiamen University, Xiamen 361000, China; liukun0309@163.com (K.L.); bioods@163.com (Q.Z.); mpf1314@126.com (P.M.); vivishao@foxmail.com (W.S.); luckydan100@126.com (D.L.); yws_huoyun@126.com (W.Y.); xmlinzhen@139.com (Z.L.); linqingyuan2007@163.com (Q.L.); 2 Chinese People’s Liberation Army No. 174 Clinical College, Anhui Medical University, Xiamen 361000, China; lotionmian@163.com; 3 Department of Oncology, Sichuan Academy of Medical Sciences & Sichuan Provincial People’s Hospital, Chengdu 610072, China; lanht@sina.com

In the original publication [1], there was a mistake in Figure 4B as published. During the review of our figures, we discovered that Figure 4B inadvertently contained a duplicated image due to an oversight in our data management. The corrected Figure 4 appears below. The authors state that the scientific conclusions are unaffected. This correction was approved by the Academic Editor. The original publication has also been updated.

## Figures and Tables

**Figure 4:**
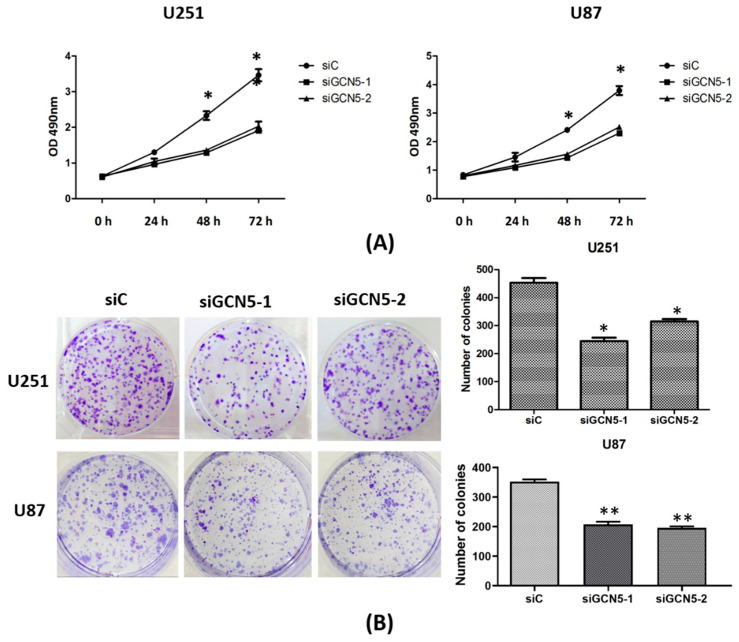
GCN5 knockdown suppressed cell proliferation and colony formation ability. (**A**) MTS assay indicated that GCN5 knockdown suppressed cell growth rate in U251 and U87 cell lines; (**B**) Colony formation assay showed that GCN5 knockdown suppressed cell colony formation ability in U251 and U87 cell lines (* *p* < 0.05; ** *p* < 0.01).
