# Supplementary material for: GCN5 Potentiates Glioma Proliferation and Invasion via STAT3 and AKT Signaling Pathways
Source: Int J Mol Sci. 2015 Sep 10;16(9):21897–910. doi: 10.3390/ijms160921897 (PMC4613287; doi:10.3390/ijms160921897)
Supplement: Supplementary file 1 [file ijms-16-21897-s001.pdf]

Supplementary Information

Table S1. Characteristics of Glioma patients

Patient Number	Age	Sex	WHO Classification	Pathological Diagnosis
1	41	Male	II	Oligodendrocyte
2	51	Female	IV	Glioblastoma
3	43	Male	II	Astroglioma
4	38	Male	IV	Glioblastoma
5	45	Female	II	Astroglioma
6	75	Female	III	Anaplastic astrocytoma
7	14	Female	IV	Glioblastoma
8	39	Male	I	Pilocytic astrocytoma
9	44	Male	IV	Glioblastoma
10	52	Female	II	Oligodendrocyte
11	45	Female	IV	Glioblastoma
12	55	Male	I	Pilocytic astrocytoma
13	58	Female	IV	Glioblastoma
14	53	Male	II	Astroglioma
15	52	Male	III	Anaplastic astrocytoma

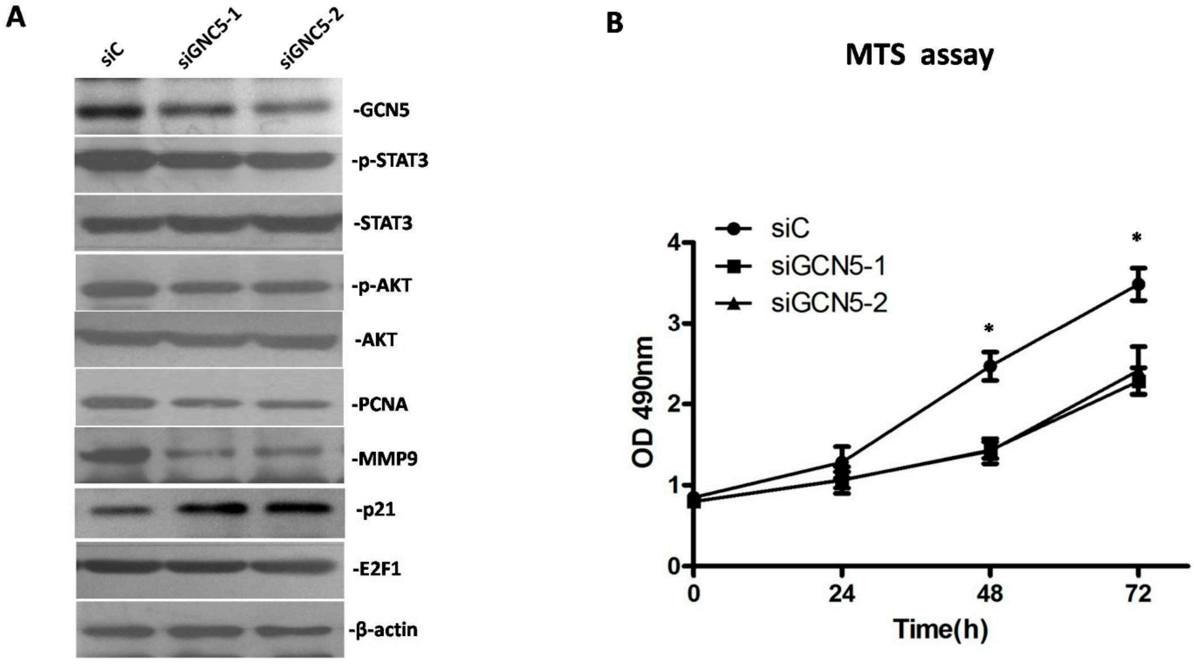

Figure S1. (**A**) GCN5 knockdown decreased the expression of p-STAT3, p-AKT, PCNA, MMP9 and increased the expression of p21 in CHG5 cell; and (**B**) MTS assay indicated that GCN5 knockdown suppressed cell growth rate in CHG5 cell (* $p < 0.05$).
